# The NF-κB Subunit RelA/p65 Is Dispensable for Successful Liver Regeneration after Partial Hepatectomy in Mice

**DOI:** 10.1371/journal.pone.0046469

**Published:** 2012-10-01

**Authors:** Marc Ringelhan, Roland M. Schmid, Fabian Geisler

**Affiliations:** 2nd Medical Department, Klinikum rechts der Isar, Technical University of Munich, Munich, Germany; Chinese University of Hong Kong, Hong Kong

## Abstract

**Background:**

The transcription factor NF-κB consisting of the subunits RelA/p65 and p50 is known to be quickly activated after partial hepatectomy (PH), the functional relevance of which is still a matter of debate. Current concepts suggest that activation of NF-κB is especially critical in non-parenchymal cells to produce cytokines (TNF, IL-6) to adequately prime hepatocytes to proliferate after PH, while NF-κB within hepatocytes mainly bears cytoprotective functions.

**Methods:**

To study the role of the NF-κB pathway in different liver cell compartments, we generated conditional knockout mice in which the transactivating NF-κB subunit RelA/p65 can be inactivated specifically in hepatocytes (*Rela^F/F^AlbCre*) or both in hepatocytes plus non-parenchymal cells including Kupffer cells (*Rela^F/F^MxCre*). 2/3 and 80% PH were performed in controls (*Rela^F/F^*) and conditional knockout mice (*Rela^F/F^AlbCre* and *Rela^F/F^MxCre*) and analyzed for regeneration.

**Results:**

Hepatocyte-specific deletion of RelA/p65 in *Rela^F/F^AlbCre* mice resulted in an accelerated cell cycle progression without altering liver mass regeneration after 2/3 PH. Surprisingly, hepatocyte apoptosis or liver damage were not enhanced in *Rela^F/F^AlbCre* mice, even when performing 80% PH. The additional inactivation of RelA/p65 in non-parenchymal cells in *Rela^F/F^MxCre* mice reversed the small proliferative advantage observed after hepatocyte-specific deletion of RelA/p65 so that *Rela^F/F^MxCre* mice displayed normal cell cycle progression, DNA-synthesis and liver mass regeneration.

**Conclusion:**

The NF-κB subunit RelA/p65 fulfills opposite functions in different liver cell compartments in liver regeneration after PH. However, the effects observed after conditional deletion of RelA/p65 are small and do not alter liver mass regeneration after PH. We therefore do not consider RelA/p65-containing canonical NF-κB signalling to be essential for successful liver regeneration after PH.

## Introduction

The liver has a unique capacity to regenerate after resection. In the mouse model of 2/3 partial hepatectomy (2/3 PH) a sequence of well orchestrated cellular events is initiated which leads to proliferation of the normally quiescent organ to ultimately restore liver function and size within 7–10 days [1]–[3]. In the mouse DNA synthesis of the remaining hepatocytes peaks at about 36–42 hours after PH. To get prepared for cell cycle entrance, multiple signalling pathways are activated within the first hours after PH, which has traditionally been denoted the “priming phase” [1], [3]. Among others the transcription factor NF-κB consisting of the subunits RelA/p65 and p50 was early identified to be quickly activated after PH within 30 minutes [4], the functional relevance of which is still a matter of debate.

In canonical NF-κB-signalling, RelA/p65-p50 is the prototypical NF-κB heterodimer to regulate transcription of genes that control inflammation, cell death, and proliferation. RelA/p65 is kept inactive in the cytoplasm bound to its inhibitor IκB, which is under the control of the IKK complex, consisting of the catalytic subunits IKKα (IKK1), IKKβ (IKK2) and the regulatory subunit IKKγ (NEMO). Upon stimulation by cytokines such as TNF, IκB is phosphorylated and degraded after ubiquitination thereby unmasking a nuclear localisation sequence (NLS) of RelA/p65 ultimately resulting in nuclear translocation and transcriptional activity of NF-κB [5], [6].

First attempts to inactivate NF-κB signalling in rodent models to unravel its function in liver regeneration suggested intact NF-κB signalling to be crucial for normal PH-induced regeneration. The adenoviral transfer of a non-degradable NF-κB superrepressor (AdIκBα) inhibiting nuclear translocation of RelA/p65 and NF-κB activation within all liver cells led to liver apoptosis and impaired hepatocyte proliferation in rat and mouse [7], [8]. In contrast, attenuation of NF-κB activity specifically in about 45% of hepatocytes by conditional expression of an IκBα superrepressor (ΔN-IκBα) under the control of the transthyretin promoter did not alter liver regeneration after PH in mice [9]. Furthermore, conditional hepatocyte-specific deletion of IKKβ in *Ikkβ^F/F^AlbCre* animals was reported not to alter PH-induced hepatocyte proliferation [10]. However, the same group found liver regeneration to be impaired, when *Ikkβ* was inactivated in all liver cells including Kupffer cells in *IKKβ^F/F^MxCre* mice [11]. Taken these results together, a concept has evolved that supports NF-κB signalling to be critical especially within non-parenchymal cells to drive an adequate early cytokine response important for normal regeneration after PH. According to this concept, NF-κB signalling within hepatocytes would be irrelevant for a proper regenerative response but rather fulfill a cytoprotective role after PH [7]–[11]. However, recently it was shown that hepatocyte-specific inactivation of IKKβ in *Ikkβ^F/F^AlfpCre* [12] or *Ikkβ^F/F^AlbCre* animals [13] rather accelerates cell cycle progression while pharmacological systemic inhibition of IKKβ did not alter liver regeneration after PH [12]. From previous studies we have learned, that conditional deletion of the IKKβ subunit of the IKK complex does not completely block but rather attenuates NF-κB activation [10], [14], [15]. We therefore asked whether discrepancies in previous studies investigating NF-κB in liver regeneration could be attributed to different degrees of inhibition of the NF-κB in the models used. Therefore, we used a conditional knockout mouse model in which the transactivating NF-κB subunit RelA/p65 which is essential for canonical NF-κB activation can be inactivated either specifically in hepatocytes (*Rela^F/F^AlbCre*) or in all liver cells (*Rela^F/F^MxCre*). Here, we report that genetic inactivation of RelA/p65 within hepatocytes does not lead to enhanced liver injury or apoptosis nor alter liver mass regeneration after 2/3 or extended PH even though cell cycle progression is accelerated. Furthermore, when RelA/p65 is inactivated in all liver cells including Kupffer cells we found an altered early cytokine response after PH. However, this only equalized the accelerated cell cycle progression observed after hepatocyte-specific deletion of RelA/p65 but did not significantly impair liver mass regeneration. Taken together, our data support a concept in which canonical NF-κB-signalling serves certain modulating but opposite functions within parenchymal and non-parenchymal liver cells after PH. However, normal liver mass regeneration occurs successful in livers lacking RelA/p65.

## Materials and Methods

### Mice

Generation of conditional RelA/p65-knockout animals (*Rela^F/F^*) was described previously [15], [16]. Shortly, in these mice exons 7 to 10 of the *Rela* gene are flanked by loxP sites leading to the generation of a truncated and functionally inactive RelA/p65 protein (Δp65) that lacks the Rel Homolgy Domain (RHD) upon *Cre*-mediated recombination. *Rela^F/F^* animals were crossed with *AlbCre* or *MxCre* animals [17], [18] to generate *Rela^F/F^AlbCre* or *Rela^F/F^MxCre* mice as described [15]. In *Rela^F/F^AlbCre* animals *Rela* is inactivated specifically in hepatocytes and biliary cells but not in non-parenchymal liver cells during late embryonic development [19], [20]. In contrast, recombination of *floxed* alleles was achieved in *Rela^F/F^MxCre* animals by a single i.p. injection of poly(I)-poly(C) (10 µg/g body weight) which leads to inactivation of *Rela* in IFNα-responsive tissues most efficiently in the liver including both hepatocytes and non-parenchymal cells [11], [15], [18]. All experiments were performed according to the protocols approved by our Institutional Animal Care and veterinarian office of the State of Bavaria (*Regierung von Oberbayern*, approval ID: TVA 55.2-1-54-2531-55-07) according to the National Institutes of Health “Guide for the Care and Use of Laboratory Animals”.

### Partial Hepatectomy

Partial hepatectomy was performed in age- (8–10 weeks) and sex-matched animals using inhalation anaesthesia with isoflurane (2% isoflurane, O_2_ 2 l/min). For 2/3 PH the left lobe (LL), the left median lobe (LML) and right median lobe (RML) were each ligated separately and resected with taking care not to injure the gallbladder (Fig. S1). For 80% PH the lower right lobe (LRL) and one omental lobe (OL) were additionally removed. Resected lobes served as controls (PH 0h) for biochemical analysis.

### Tissue Processing and Analysis of Liver Regeneration after PH

Animals were injected i.p. with BrdU 100 µg/g BW two hours before they were sacrificed at the indicated time points after PH. Blood was withdrawn from the inferior caval vein, centrifuged, and serum was kept at −80°C until assayed. Liver tissue was processed for histology or snap-frozen and stored at −80°C. For determination of the extent of liver injury, ALT serum levels were determined by standard procedures. TNF and IL-6 serum levels were determined using a commercially available murine quantitative enzyme-linked immunosorbent assay (ELISA) kit (R&D Systems). Liver mass regeneration (%) at the respective time points after PH was determined by estimating original liver mass form the mass of the resected lobes at the time of PH. For this calculation the resected lobes were assumed to make up 67% and 78% in 2/3 PH and 80% PH respectively. These proportions had been determined in more than 20 mice of the same genetic background.

### Kupffer Cell Isolation

To isolate liver Kupffer cells (KC) in order to verify efficient deletion of RelA/p65 on protein level, liver cells were isolated by retrograde Collagenase-perfusion as described previously [15]. The non-parenchymal cell fraction was pelleted by centrifugation (350 G, 10 min 4°C) and layered on top of a preformed two-step Percoll-gradient (50% and 25%). After centrifugation (800 G, 20 min, 4°C) cells in the KC-interphase were collected, pelleted, and resuspended in RPMI-1640 Medium (Sigma) at 0.5×10^6^ cells/ml and selective adherence of KC was allowed for 15 min.

### Protein Isolation and Immunoblot Analysis

For preparation of whole-cell protein extracts livers or cells were homogenized in Triton-lysis buffer (1% Triton, 20 mM Tris-HCl, 150 mM NaCl, 1 mM Na_2_EDTA, 1 mM EGTA, 1 mM PMSF and protease- and phosphatase-inhibitor cocktail). The lysate was sonicated and clarified by centrifugation, snap-frozen in liquid nitrogen and stored at −80°C until assayed. Protein extracts were analyzed by discontinuous SDS-PAGE as described previously [15]. Antibodies used were: rabbit anti-p65, anti-PCNA, anti-β-actin, anti-Cyclin A, anti-Cyclin D1 (all Santa Cruz), anti-JNK, anti-phospho-JNK, anti-STAT, anti-phospho-STAT (all Cell Signaling).

### Histology

For histological analysis livers were fixed in 4% neutral phosphate-buffered paraformaldehyde for 20 hours, embedded in paraffin, and sectioned. Serial 3.5 µm-thick sections were stained with H&E using a standard protocol and evaluated under light microscopy. For immunohistochemical analysis tissues were processed as described previously [19] using anti-BrdU (Serotec) and goat anti-p65 (Santa Cruz). The TUNEL assay was performed using the in situ cell death detection kit POD (Roche Diagnostics Corp.) according to the manufacturer’s instructions. BrdU-uptake (%) was quantified by counting BrdU positive and negative hepatocyte nuclei in 10 random ×200-power fields in two different liver lobes.

### Real-time PCR

Total RNA from livers was extracted using the RNeasy kit (Qiagen, Germany) according to the manufacturer’s instructions. RNA was transcribed into cDNA using random hexamers and the TaqMan Reverse Transcription Kit (Applied Biosystems). cDNA was further analyzed by real-time PCR as described previously [16] on an ABI 7700 Sequence Detection System (Applied Biosystems). All samples were analyzed in triplicate and data were calculated by 2-ΔΔCt method as described by the manufacturer and were expressed as fold increase over controls as indicated in the figure legends. Cyclophillin expression was used for normalization (primer sequences can be obtained by the authors upon request).

#### Electrophoretic mobility shift assay (EMSA)

For preparation of nuclear enriched whole-cell protein extracts snap frozen livers were homogenized in CelLytic™-Buffer and a high salt buffer (NuCLEAR™ Extraction Kit, Sigma-Aldrich). In brief, snap frozen liver samples were homogenized and lysed in isotonic buffer (10 mM Tris-HCl, pH 7.5, 2 mM MgCl2, 3 mM CaCl2, 0.5 M sucrose, 1 mM DTT, and protease inhibitor mixture) on ice for 20 min, and 0.6% IGEPAL CA-630 solution was added followed by centrifugation at 9000×g for 10 min. The nuclear pellet was resuspended in extraction buffer (20 mM HEPES, pH 7.9, 1.5 mM MgCl2, 0.42 M NaCl, 0.2 mM EDTA, 25% glycerol, 1 mM DTT, and protease inhibitor mixture) on a vortex mixer for 30 min at 4°C, sonicated on ice and centrifuged at 20000×g for 5 min. The supernatants were used as nuclear enriched whole-cell protein extracts. For EMSA 7,5 µg protein was assembled with 1 µl 10× Binding Buffer (100 mM Tris, 500 mM KCl, 10 mM DTT; pH 7.5), 2 µl of 50 mM DTT, 2.5% Tween-20, 1 µl 1% NP-40, 12 µl ddH_2_O, 1 µg of Poly (dI.dC) (1 µg/µl in 10 mM Tris, 1 mM EDTA; pH 7.5, Sigma-Aldrich) as nonspecific competitor and incubated with 1 µl of IRdye 700 pre-labeled NF-κB consensus oligonucleotide 50 fmol/µl (LI-COR) in dark for 30 min at room temperature. Cold competition was performed in the presence of 100-fold excess non-labeled consensus oligonucleotides respectively for 10 minutes prior to the addition of labeled oligonucleotides. The samples were loaded with 1 µl of 10X Orange Loading Dye on a 8% pre-run TBE-polyacrylamid gel and electrophoresis was continued at 80 V for 90–120 minutes. The signal was then detected and quantified with Odyssey infrared imaging system (Li-COR).

### Statistics

Data are expressed as mean ± standard error (SEM). Differences between groups were analyzed by Student’s t-test where appropriate. In all cases, sample sizes (as indicated in the figures) were chosen to produce statistically unambiguous results. A *P* value of 0.05 or less was considered significant.

## Results

### Hepatocyte-specific Loss of RelA/p65 does not Result in Enhanced Liver Damage after 2/3 PH

**Figure 1:**
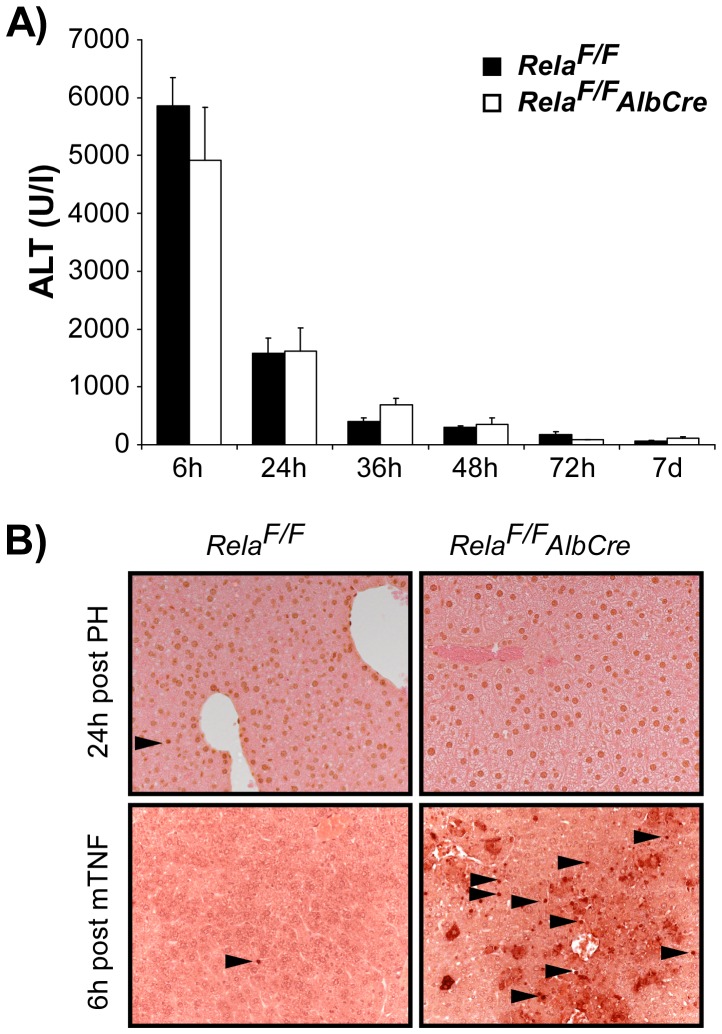
Hepatocyte-specific inactivation of RelA/p65 in *Rela^F/F^AlbCre* animals does not lead to enhanced liver injury after 2/3 PH. (**A**) *Rela^F/F^* and *Rela^F/F^AlbCre* animals were subjected to 2/3 PH and ALT serum levels were determined at the indicated time points. Data are expressed as mean ± SEM (n = 3−6 animals per time point and group). (**B**) No significant apoptosis was detected at any time point after PH in control or mutant mice as assessed by TUNEL-staining. Representative TUNEL stainings from control and mutant livers harvested 24 h post PH are shown in the upper row. Liver sections from TNF-injected (i.v. 10 ng/g BW) control and *Rela^F/F^AlbCre* animals which are highly sensitive to TNF-induced apoptosis served as control (lower panel, magnification ×200).

To study the functional role of the transactivating NF-κB subunit RelA/p65 in liver regeneration, *Rela^F/F^AlbCre* animals were subjected to 2/3 PH. Livers of these mice lack a functional RelA/p65 specifically in hepatocytes and loss of NF-κB binding activity upon LPS- or TNF-stimulation renders them highly sensitive to TNF-induced apoptosis [15]. Because PH is known to trigger a cytokine response with significant TNF-serum levels [21], we were curious whether the loss of RelA/p65 within hepatocytes would lead to enhanced liver injury after PH. To our surprise, we did not observe significantly different ALT levels in *Rela^F/F^AlbCre* animals as compared to control animals when followed over 7 days after 2/3 PH (Fig. 1A). Moreover, tissue integrity was not disturbed and no significant apoptotic cell death could be detected at any time point as assessed by histology and TUNEL-staining (Fig. 1B). Overall mortality after 2/3 PH did not differ significantly between groups (<5%) and could be attributed to technical complications during surgery. These results suggest that the main NF-κB subunit RelA/p65 is not essential to protect hepatocytes form cell death after 2/3 PH.

### Hepatocyte-specific Inactivation of RelA/p65 Causes an Accelerated Cell Cycle Progression without Altering Liver Mass Regeneration after 2/3 PH

**Figure 2:**
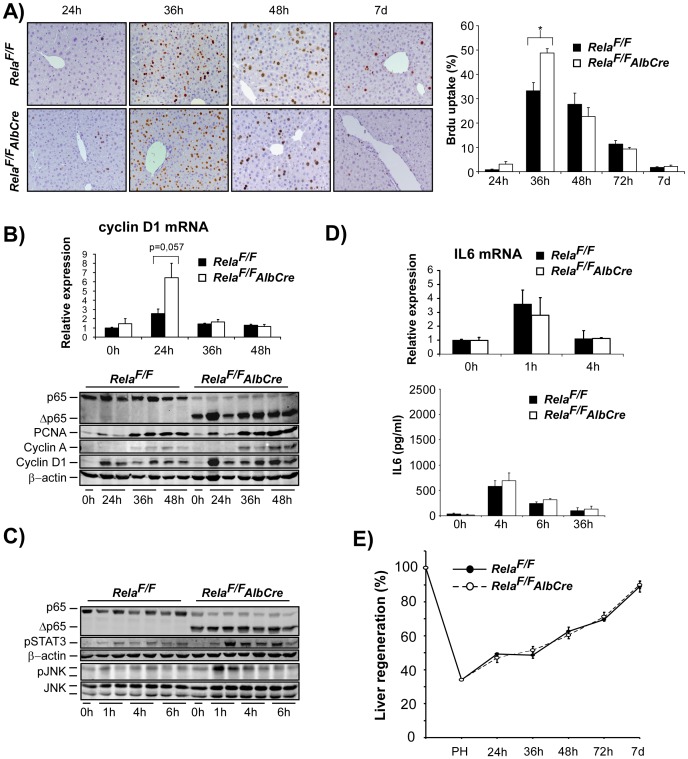
Hepatocyte-specific inactivation of RelA/p65 results in accelerated cell cycle progression without altering liver mass regeneration after 2/3 PH. (**A–E**) 2/3 PH was performed in *Rela^F/F^AlbCre* and *Rela^F/F^* control animals. (**A**) DNA synthesis was assessed by hepatocyte BrdU labelling at the indicated time points and quantified as described in *Methods* (representative×200 anti-BrdU stained liver sections are shown in the left panel, n = 4−8 animals per time point and group were counted for quantification, right panel). (**B**) Accelerated cell cycle progression in *Rela^F/F^AlbCre* mice as determined by mRNA levels of Cyclin D1 24 h post PH (upper panel) and WB analysis of cell-cycle associated proteins (PCNA, Cyclin A, Cyclin D1). Lysates were controlled for effective deletion of WT-RelA/p65 and β-actin served as loading control. Two representative lysates from each time point post PH are shown. (**C**) Loss of hepatocyte RelA/p65 leads to enhanced phosphorylation of STAT3 and JNK during the first hours after PH as assessed by immunoblot analysis (upper panels). (**D**) Liver IL-6 mRNA induction as determined by RT-PCR (upper graph) and IL-6 serum levels (ELISA, lower graph) were not different between groups. (**E**) Liver mass regeneration (%) determined as described in *Methods* did not differ between the groups. Data from BrdU-labelling, cytokine analysis, RT-PCR, and analysis of liver mass regeneration are presented as the average ± SEM for 3–6 animals per time point per group. *, p≤0.05 for mutant vs. control mice.

We next asked, whether the regenerative response would be altered in *Rela^F/F^AlbCre* animals after 2/3 PH as assessed by BrdU uptake as a measure for DNA-synthesis. Controls and mutant mice displayed similar kinetics of BrdU-uptake, however, the onset of BrdU uptake started earlier in *Rela^F/F^AlbCre* animals with significantly higher levels at 36 hours after PH (Fig. 2A). Consistent with histological findings, G1/S-phase-specific Cyclin D1 mRNA expression at 24 hours after PH was higher in *Rela^F/F^AlbCre* animals as was Cyclin D1 expression on protein level at 24–48 hours after PH (Fig. 2B). Moreover, WB analysis showed more pronounced PCNA and Cyclin A expression at 36 and 48 hours post PH. Theses findings suggest that hepatocyte loss of a functional RelA/p65 leads to an accelerated cell cycle progression after PH. As JNK signalling is suppressed by NF-κB signals [10], [15], [22] and Cyclin D1 expression is known to be regulated by JNK/AP1 [23], we analyzed early phosphorylation of JNK during the priming phase at 1, 4 and 6 hours after PH. While control animals did not display significant phospho-JNK levels, phosphorylation of p54-JNK was clearly induced in liver lysates from *Rela^F/F^AlbCre* after PH. Furthermore, we found enhanced levels phospho-STAT3 in livers of *Rela^F/F^AlbCre* animals as compared to controls (Fig. 2C). However, serum levels of the gp130 ligand and inducer of the JAK/STAT signalling pathway IL-6 or induction of IL-6 mRNA were not significantly different between the groups (Fig. 2D), indicating and confirming that NF-κB signals within hepatocytes negatively regulate STAT3 activation [24]. Of note, we were unable to detect any TNF-serum levels by ELISA in sera or liver lysates of control or mutant mice at 1, 4, 6 or 12 hours after PH (data not shown). mRNA expression levels of TNF were hardly detected in liver samples (CT values >31) and not different in *Rela^F/F^AlbCre* livers at 1 and 4 h after PH (data not shown).

Despite the above findings indicating a slight but robust acceleration of cell cycle progression in RelA/p65 deficient hepatocytes, the time course of liver mass regeneration was not different between groups and all animals almost fully reached the initial liver mass within 7 days after 2/3 PH irrespective of the genotype (Fig. 2 E).

### Extended Liver Mass Resection (80% PH) does not Lead to Impaired Regeneration or Increased Liver Cell Damage after Hepatocyte-specific Inactivation of RelA/p65

**Figure 3:**
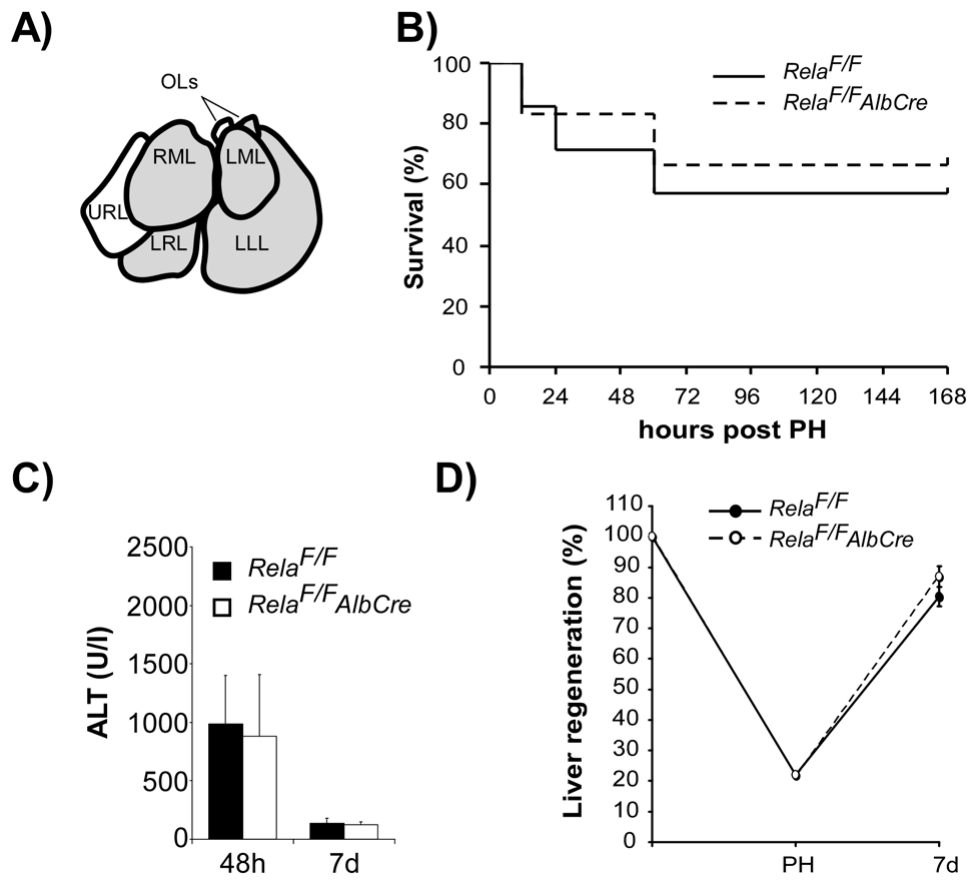
Extended liver mass resection of 80% does not lead to impaired regeneration or increased liver cell damage after hepatocyte-specific inactivation of RelA/p65. (**A**) For extended (80%) PH the lower right lobe (LRL) and one omental lobe (OL) were additionally resected to standard 2/3 PH in control and *Rela^F/F^AlbCre* animals. (**B**) Survival of control and mutant animals did not differ between groups and was 4/6 in *Rela^F/F^* and 4/7 in *Rela^F/F^AlbCre* animals. ALT levels determined at 48 hours (**C**) and liver mass regeneration at 7 days post 80% PH (**D**).

Our results are unexpected insofar as the main NF-κB subunit RelA/p65 is not fundamentally essential within hepatocytes to preserve liver cell integrity after 2/3 PH. To test whether this holds true also for extended liver mass resection, controls and *Rela^F/F^AlbCre* animals were subjected to extended partial hepatectomy. Additionally to the left and median lobes, the lower right lobe and the ventral omental lobe were resected resulting in about 80% PH (Fig. 3A) which is reported to be lethal to mice in 60–70% [25]. Under these conditions however, survival of control (4/6) and *Rela^F/F^AlbCre* mice (4/7) after 80% PH (Fig. 3B) and ALT levels of surviving animals at 48 hours and 7 d after extended PH were not significantly different (Fig. 3C). Livers of those animals not surviving all died within 60 hours after extended PH and displayed extensive centrilobular necrosis lacking significant apoptosis irrespective of genotype investigated (data not shown). Finally, liver mass regeneration of surviving animals at 7 days expressed in %-regeneration (Fig. 3D) or in liver weight/body weight (%) (*Rela^F/F^* 3,4±0,2%; *Rela^F/F^AlbCre* 3,5±0,1%) did not differ significantly between genotypes.

Our results from 2/3 and 80% PH support the concept that genetic loss of intact NF-κB signalling within in the parenchymal cellular compartment of the liver does not particularly sensitize this organ to enhanced cell death after liver resection but rather leads to a certain proliferation advantage that however does not significantly alter overall restoration of the total number and mass of hepatocytes.

### Genetic Inactivation of RelA/p65 in Both Parenchymal and Non-parenchymal Cells Neutralizes the Proliferative Advantage Seen after Hepatocyte-specific Deletion of RelA/p65 and Leads to a Normal Regenerative Response after PH

**Figure 4:**
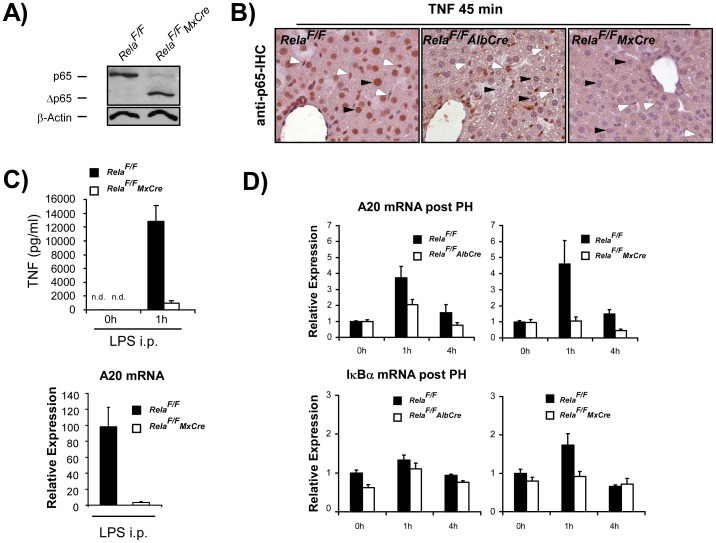
Genetic deletion of WT RelA/p65 in all liver cells including Kupffer cells impairs NF-κB dependent cytokine response and activation of NF-κB target genes in *Rela^F/F^MxCre* animals. (**A**) WT RelA/p65 is deleted in Kupffer cells isolated from *Rela^F/F^MxCre* animals 7 days after one single i.p. injection of poly-IC (10 µg/g BW) as assessed be immunoblot analysis with anti-p65. β-actin served as loading control. (**B**) 45 min after i.p. TNF (25 ng/g BW) nuclear translocation of RelA/p65 is seen in hepatocytes (black arrowheads) and non-parenchymal cells (white arrowheads) in *Rela^F/F^* controls, confined to non-parenchymal cells in *Rela^F/F^AlbCre* animals and virtually absent in all liver cells in *Rela^F/F^MxCre* animals (magnification×200). **(C)** LPS (2 mg/kg) -induced TNF-response in mouse serum (left panel) and LPS-induced induction of the NF-κB target gene *Tfnaip3* encoding A20 in the liver (right panel) is abolished in *Rela^F/F^MxCre* animals as assessed by ELISA and RT-PCR respectively. (**D**) A20 mRNA and IκBα mRNA expression as assessed by RT-PCR analysis at the indicated times post PH is inhibited in *Rela^F/F^AlbCre* animals but abolished in *Rela^F/F^MxCre* animals. Data from cytokine analysis and RT-PCR are presented as the average ± SEM for 3–6 animals per time point per group.

In contrast to previous reports that attribute PH-induced NF-κB-activation to parenchymal cells [7], [26], a more recent concept proposes that Kupffer cells are the main source of NF-κB-activation that also orchestrate the early cytokine response after PH [8], [9], [11]. Therefore, to additionally inactivate canonical NF-κB signalling in Kupffer cells, we used *Rela^F/F^MxCre* animals to study liver regeneration after PH. Effective deletion of wildtype RelA/p65 was confirmed by WB analysis of lysates from Kupffer cells isolated form control and *Rela^F/F^MxCre* animals 7 days after i.p. injection with polyIC (Fig. 4A). To verify that nuclear translocation of RelA/p65 as a prerequisite for NF-κB activation does not occur in both parenchymal and non-parenchymal cellular compartments in the livers of *Rela^F/F^MxCre* animals, *Rela^F/F^MxCre* and control animals were challenged with an i.p. injection of TNF (25 ng/g BW) and subjected to anti-p65-IHC analysis. TNF is a strong inducer of NF-κB and consequently, at 45 min after TNF-injection RelA/p65 translocation can be observed in hepatocytes and non-parenchymal cells in control animals (Fig. 4B, left). In *Rela^F/F^AlbCre* animals nuclear staining of RelA/p65 is restricted to non-parenchymal cells while nuclear staining of RelA/p65 is virtually absent in all cells in the livers of TNF-treated *Rela^F/F^MxCre* animals (Fig. 4B, middle and right panel respectively). Consequently and as previously reported, *Rela^F/F^MxCre* mice display a blunted LPS-induced TNF-response because nuclear translocation of RelA/p65 in Kupffer cells as the main source of LPS-induced NF-κB-dependent TNF-production is inhibited (Fig. 4C, left panel). Accordingly, stimulation of the NF-κB target gene A20 after LPS-challenge was abolished in *Rela^F/F^MxCre* mice (Fig. 4C, right panel). PH-induced induction of A20 was only attenuated in *Rela^F/F^AlbCre* mice but abolished in *Rela^F/F^MxCre* animals (Fig. 4D). However, PH-induced A20-mRNA induction in control animals (4-fold) was very low when compared to LPS injection (100-fold), suggesting that liver NF-κB activation is very weak after PH. Similarly, the magnitude of PH-induced induction of the NF-κB target gene IκBα in controls was mild, but completely inhibited in *Rela^F/F^MxCre* mice (Fig. 4D). The assumption that NF-κB activation was only mild after PH under our experimental conditions was confirmed by anti-p65-IHC where nuclear staining was only occasionally observed mainly in non-parenchymal cells of control animals when analyzed at 1 h and 4 h post PH (Fig. S2). Furthermore EMSA analysis for NF-κB binding activity at 1 h post PH did not give a robust signal in either genotype analyzed (Fig. S2).

**Figure 5:**
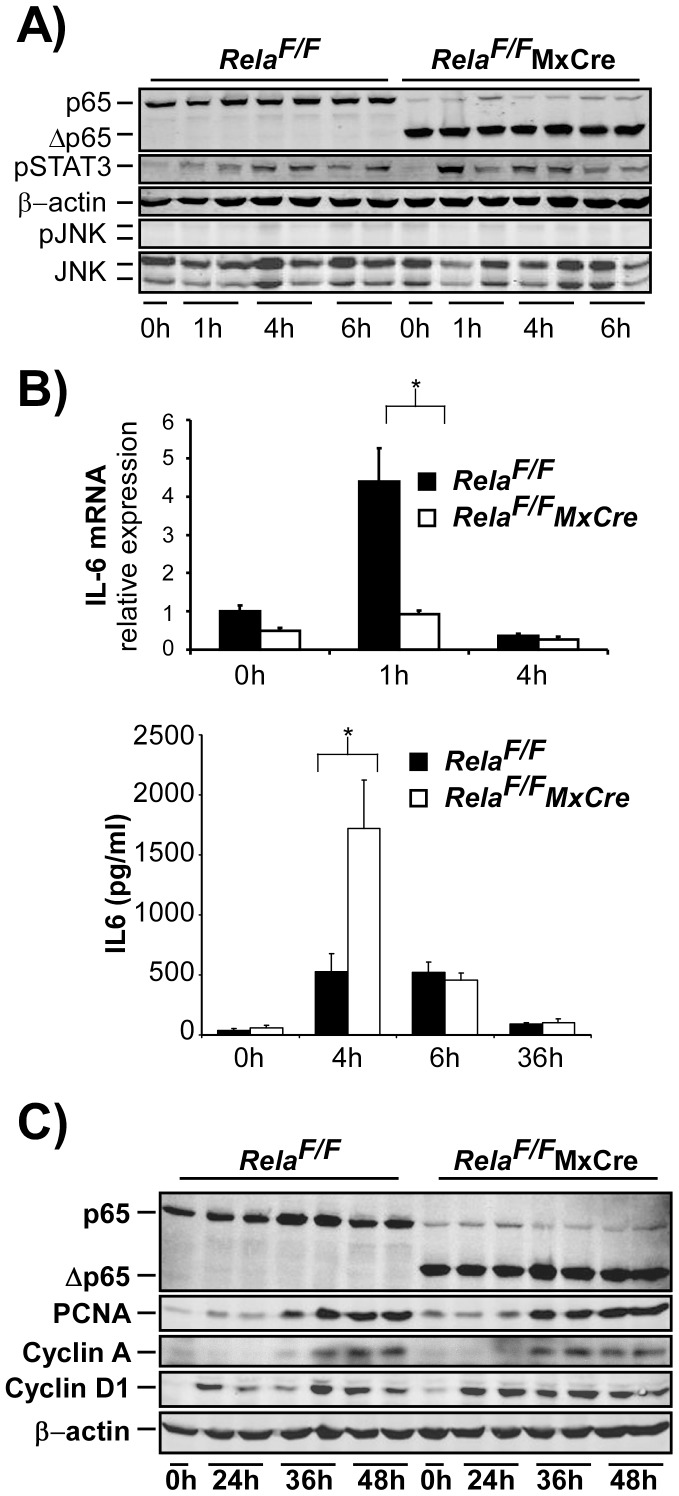
Additional genetic deletion of RelA/p65 in all liver cells in *Rela^F/F^MxCre* alters the cytokine response without significantly altering cell cycle progression after 2/3 PH. (**A–C**) 2/3 PH was performed on *Rela^F/F^* and *Rela^F/F^MxCre* animals and analyzed as indicated. (**A**) Levels of phospho-STAT3 in control and *Rela^F/F^MxCre* animals were not different while phosphorylation of JNK was barely detected in either group as assessed be immunoblot analysis. (**B**) Induction of liver IL-6 mRNA was inhibited in *Rela^F/F^MxCre* animals as determined by RT-PCR (upper image), however, IL-6 serum levels were significantly elevated in livers of *Rela^F/F^MxCre* mice at 4 h post PH (lower image). (**C**) Immunoblot analysis of cell cycle associated proteins was performed as described in Fig. 2. Data from cytokine analysis and RT-PCR are presented as the average ± SEM for 3–6 animals per time point per group. *, p≤0.05 for mutant vs. control mice.

To determine the impact of the additional loss of RelA/p65 in the non-parenchymal cellular compartment to the cytokine and regenerative response after PH, *Rela^F/F^MxCre* animals and controls were subjected to 2/3 PH. Phospho-STAT3 levels at 1 h, 4 h and 6 h after PH were not significantly different between the strains (Fig. 5A, upper and middle panel). Furthermore, phospho-JNK levels were hardly detected in either group (Fig. 5A), suggesting that the additional deletion of RelA/p65 in non-parenchymal cells has a mitigating effect on enhanced STAT3- and JNK-phosphorylation seen after hepatocyte-specific deletion of RelA/65 in *Rela^F/F^AlbCre* animals. Because deletion of RelA/p65 occurs equally efficient in hepatocytes of both *Rela^F/F^AlbCre* and *Rela^F/F^MxCre* livers, we presume that NF-κB-dependent signals from non-parenchymal cells that would initiate STAT- and JNK-signalling are lost in *Rela^F/F^MxCre* animals.

As expected, IL-6 mRNA induction within the liver was clearly inhibited in *Rela^F/F^MxCre* animals as compared to *Cre*-negative litters at 1 h after PH (Fig. 5B, upper graph). Paradoxically, serum levels of IL-6 were significantly higher in *Rela^F/F^MxCre* animals at 4 hours after PH, suggesting that extrahepatic cells are the source of systemic IL-6 levels (Fig. 5B, lower graph). Again, TNF-serum levels could not be detected at any time point after PH (data not shown).

**Figure 6:**
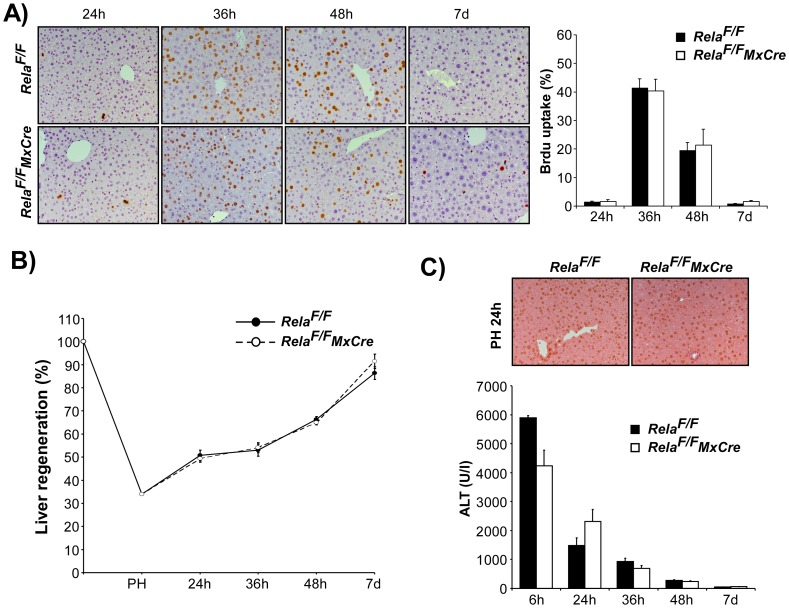
Genetic deletion of WT RelA/p65 in all liver cells in *Rela^F/F^MxCre* animals does not alter DNA-synthesis or liver mass regeneration after 2/3 PH. (**A**) DNA-synthesis as determined by BrdU-uptake and (**B**) liver mass regeneration was not different in control and mutant animals. (**C**) Representative TUNEL staining at 24 h and ALT levels did not reveal significant apoptosis or altered liver damage in *Rela^F/F^* and *Rela^F/F^MxCre* animals. Data are presented as the average ± SEM for 3–6 animals per time point per group.

Cell cycle progression was not grossly altered as assessed by immunoblot analysis of cell cycle proteins Cyclin D1, PCNA and Cyclin A in liver lysates from control and *Rela^F/F^MxCre* animals after PH (Fig. 5C). This supports the view that the additional deletion of RelA/p65 in the non-parenchymal liver cell compartment reverses the slight proliferation advantage observed after hepatocyte-specific loss of RelA/p65. Congruently, BrdU-uptake of hepatocytes was not different in *Rela^F/F^MxCre* animals (Fig. 6A) and liver mass regeneration occurred normally (Fig. 6B). No enhanced liver damage was detected in *Rela^F/F^MxCre* animals at any time after PH as determined by ALT levels and TUNEL staining (Fig. 6C).

## Discussion

The most important finding in our study is that liver regeneration after PH occurs perfectly fine when the transactivating NF-κB subunit RelA/p65 is inactivated, either in hepatocytes alone or in all liver cells. Initial studies investigating the role of NF-κB signalling in liver regeneration pointed to an essential role of NF-κB to prevent apoptotic cell death and drive proliferation after PH [7]. Harmonizing with initial studies obtained from IL-6- or TNFR1-deficient mice that were reported to display impaired liver regeneration [21], [27] an attractive model was coined where NF-κB not only serves cytoprotective functions within hepatocytes but also routes an adequate cytokine response primarily within Kupffer cells to prime hepatocytes for mitogenic stimuli to proliferate [28].

There is the current conception, that the degree of NF-κB inhibition determines the sensitivity of hepatocytes to apoptotic cell death [29], [30]. This view has been deduced from genetic studies investigating hepatocytes deficient for the IKKβ-subunit of the IKK-complex that are still capable to display residual NF-κB activity and show only mild TNF-induced cell death [10], [14], [15]. In contrast, in hepatocytes lacking the IKK-subunit IKKγ- or the NF-κB-subunit RelA/p65, TNF-induced NF-κB-activation is completely abolished rendering these cells highly sensitive to minute amounts of TNF [14], [15]. Conditional hepatocyte-specific deletion of IKKβ rather accelerates regeneration after 2/3 PH [11], [12], however, massive apoptosis and strongly impaired regeneration associated with high mortality is observed after PH in mice after hepatocyte-specific inactivation of IKKγ [31]. With respect to this latter congress report our results are unexpected insofar as we did not find any significant apoptosis after 2/3 PH suggesting that TNF levels after PH do not suffice to trigger cell death even when canonical NF-κB signalling is shut down in hepatocytes. Because RelA/p65- and IKKγ-deficient hepatocytes are highly sensitive to TNF-induced apoptosis [15], [32] but regeneration after PH is obviously different, it seems that the IKK-complex preserves cell integrity also by regulating pathways independent of canonical NF-κB signalling [33]. However, we cannot rule out that NF-κB dimers lacking RelA/p65 at later time points during liver regeneration not analyzed for NF-κB activation may be capable to induce survival signals in *Rela^F/F^AlbCre* animals. Furthermore, as pointed out below, different surgical PH-techniques may lead to different cytokine levels that might account for the differences.

Livers from *Rela^F/F^AlbCre* showed an earlier onset of cell cycle progression and a higher maximum of DNA-synthesis after PH. This was preceded be enhanced JNK-activation known to be important to drive transition from G0 to G1 as a possible mechanism for the accelerated cell cycle progression. Our findings are in agreement with results obtained from *IKKβ^F/F^AlfpCre* [12] and *IKKβ^F/F^AlbCre* [13] animals and suggest that the loss of NF-κB signals within hepatocytes goes along with a proliferation advantage after PH possibly due to an altered acute phase response or differential NF-κB-dependent control of STAT3 activity [24] Enhanced proliferation observed in *Rela^F/F^AlbCre* animals was robust but rather small and liver mass regeneration was not altered in *Rela^F/F^AlbCre* animals. So, we are reluctant to over-interpret its functional meaning in liver regeneration after PH and our results rather underscore the view that classical canonical NF-κB signalling containing RelA/p65 within hepatocytes is dispensable for liver regeneration after PH.

The small proliferation advantage of RelA/p65-deficient hepatocytes after PH was lost when RelA/p65 was inactivated in all liver cells in *Rela^F/F^MxCre* mice consistent with the popular view that NF-κB-dependent signals from non-parenchymal cells that promote regeneration are lost [1], [3]. However, considering the decisive role that is attributed to canonical NF-κB signalling in non-parenchymal liver cells in liver regeneration after PH, it is stunning that regeneration in *Rela^F/F^MxCre* animals occurs perfectly fine. We are well aware that the *MxCre*-mouse line has limitations because *MxCre*-induced deletion is not restricted to liver cells but also occurs in the spleen and other IFN-sensitive tissues [18]. Deletion of IKKβ or RelA/p65 using *MxCre* mice has been reported to alter cytokine processing in myeloid cells [34]. In this context it is possible that even though liver IL-6 mRNA is suppressed in *Rela^F/F^MxCre* animals after PH, enhanced IL-6 production from extrahepatic tissues as observed in our study might compensate for reduced IL-6 production in RelA/p65-deficient Kupffer cells. Nevertheless, Maeda et al. reported a strongly reduced regenerative response in *IKKβ^F/F^MxCre* animals [11] while systemic pharmacological inhibition of IKKβ was not found to alter liver regeneration after PH by others [12].

It is conspicuous that studies investigating the role of NF-κB signalling and cytokines in mouse liver regeneration after PH often produce conflicting results: Significant NF-κB activation in response to 2/3 PH is not found by all investigators [25] and the time point when maximal NF-κB activation is observed after PH varies from 30 minutes [4] to 12 hours after PH [8]. In our hands, the magnitude of NF-κB activation in control animals was too low to be detected using NF-κB-binding assays, and we only observed sparse nuclear translocation of RelA/p65 mainly in non-parenchymal cells with immunohistochemistry at 1 h post PH (Fig. S2). Furthermore, TNF-serum levels are only infrequently detected by different research groups and the effect of genetic deletion or inhibition of TNF or its receptor TNFR1 has produced clearly different results ranging from “grossly impaired liver regeneration and high lethality” to “no functional relevance” after PH [21], [35], [36]. Also, while initial studies in *Il-6* null mice suggested IL-6 to be essential for both proliferation and survival [27], subsequent studies in *Il-6* null mice or in mice with ablation of the IL-6 downstream signalling molecule gp130 showed essentially normal regeneration and clearly relativized the importance of IL-6 in regeneration after PH [37], [38]. Remarkably, also genetic deletion of the TLR-adaptor protein MYD88 has revealed conflicting results by different groups [39], [40]. Though both groups reported a blunted IL-6 and TNF-response in *Myd88* null mice, only one group found liver regeneration to be impaired [39]. Furthermore, in a recent study a dramatic influence of different puncture sites for blood sampling on IL-6 levels was pointed out. In that study, even an anti-proliferative effect of IL-6 on liver regeneration after PH was suggested [41]. It is unlikely that all these contradictory findings from many different laboratories can all be attributed to differences in genetic background or breeding conditions of the animals.

2/3 PH is considered the golden standard to study liver regeneration in mice. The more it is surprising that the surgical procedure to perform PH has not been standardized. The original mass ligation of the median and the left liver lobe as originally described for the rat by Higgins and Anderson back in 1931 [42] has been adopted by multiple laboratories to study liver regeneration in the mouse though it has early been recognized that distinct anatomical differences such as the presence of a gallbladder in the mouse may lead to considerable mortality and less reproducible results [43]–[45]. Only infrequently separate ligation and separate resection of the liver lobes (either with or without resection of the gallbladder) have been applied in mouse studies investigating liver regeneration after 2/3 PH and only recently efforts were undertaken by researchers to provide a standard for the surgical procedure in the mouse [46]–[48]. It has been pointed out that selective ligation and resection of each liver lobe is crucial to avoid venous obstruction and consecutive necrosis that interferes with liver regeneration as sometimes observed after mass ligation [47]. Several studies suggest that the surgical technique in fact may have a strong impact on liver regeneration after PH. For instance, lethality of *Il-6* null animals was initially found to be very high [27], [49] but was absent when the lobes were resected separately and the gallbladder was left undisrupted [37]. Another striking example for the significance of the surgical procedure comes from one group that earlier found 80% lethality in hepatocyte-specific knockout mice for the HGF-receptor c-Met using the method of Higgins & Anderson [50]. However, lethality was absent when the gallbladder was left intact and the liver lobes were ligated separately [51]. It may well be the case that i.e. the magnitude of endotoxin translocation from the gut or the release from other factors that regulate the cytokine and acute phase responses as well as the magnitude of NF-κB activation are influenced by a variable extent of tissue damage caused by different surgical techniques. In initial experiments we found considerable lethality and impaired liver DNA-synthesis in *Rela^F/F^MxCre* animals when using mass ligation including cholecystectomy. This would support the notion that NF-κB dependent signals or pathways in fact may be vital to face an enhanced acute phase response and enable liver regeneration when less gentle surgical techniques are applied. However, using the surgical technique as described, we show that the transactivating NF-κB subunit RelA/p65 fulfils rather minor albeit opposite functions in parenchymal and non-parenchymal liver cells. We conclude that classical canonical NF-κB signalling containing RelA/p65 within liver cells is of minor importance for successful liver regeneration after PH. One has to be cautious regarding potential side effects of NF-κB inhibitors in the setting of inflammation. However, translating our findings to the clinical situation, it may be save to perform liver resection even in the setting of systemic (i.e. pharmacological) NF-κB inhibition.

## Supporting Information

Figure S1 **Surgical procedure for 2/3 PH. (A)** For 2/3 PH the left lower lobe (LLL), the left median and the right median lobe (LML and RML) are removed. **(B)** Each lobe is separately ligated (a-c) with taking special care not to disrupt the gallbladder (d).
(TIF)

Figure S2 **NF-κB activation after 2/3 PH. (A)** NF-κB activation in livers of indicated genotypes as assessed by anti-p65 IHC at 1 h and 4 h post PH. Nuclear staining is occasionally observed in non-parenchymal cells (white arrowheads) only in control and *Rela^F/F^AlbCre* animals at 1 h and to a lesser extent at 4 h post PH. Nuclear staining for p65 is almost never detected in hepatocytes of either genotype (black arrowhead marks a positive hepatocyte in a control animal). Nuclear staining was nearly completely absent in *Rela^F/F^MxCre* animals both in hepatocytes and non-parenchymal cells (magnification×200). **(B)** No robust NF-κB binding activity could be detected by EMSA analysis 1 h post PH in livers of indicated genotypes. Liver lysates from LPS-treated animals served as positive control (LPS 2 mg/kg i.p.).
(TIF)
