# Supplementary figures and images for: The NF-κB Subunit RelA/p65 Is Dispensable for Successful Liver Regeneration after Partial Hepatectomy in Mice
Source: PLoS One. 2012 Oct 1;7(10):e46469. doi: 10.1371/journal.pone.0046469 (PMC3462179; doi:10.1371/journal.pone.0046469)

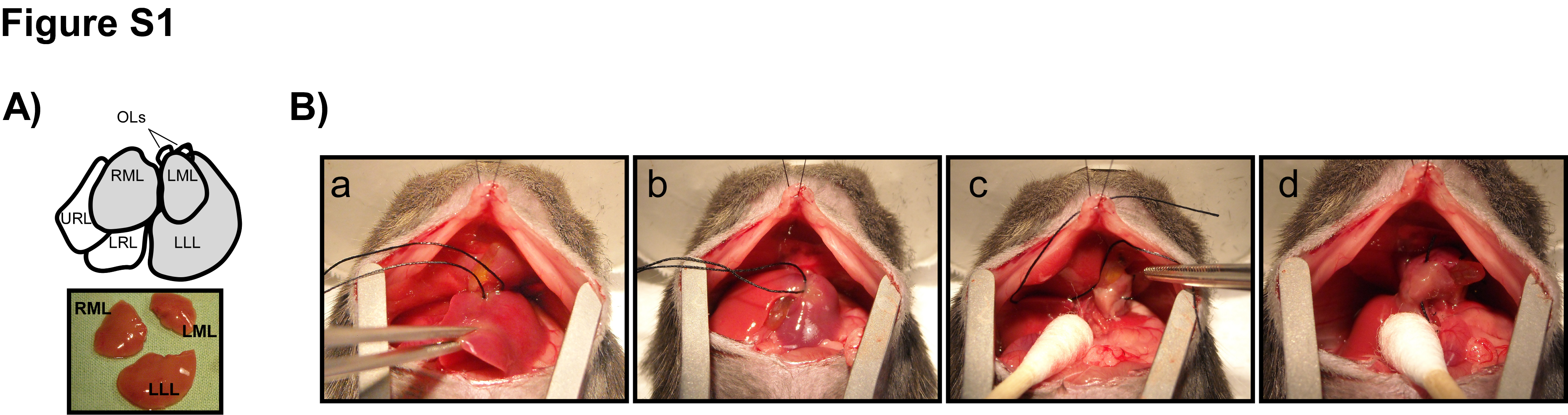

Supplement: Figure S1 — Surgical procedure for 2/3 PH. (A) For 2/3 PH the left lower lobe (LLL), the left median and the right median lobe (LML and RML) are removed. (B) Each lobe is separately ligated (a-c) with taking special care not to disrupt the gallbladder (d). (TIF) [file pone.0046469.s001.tif]

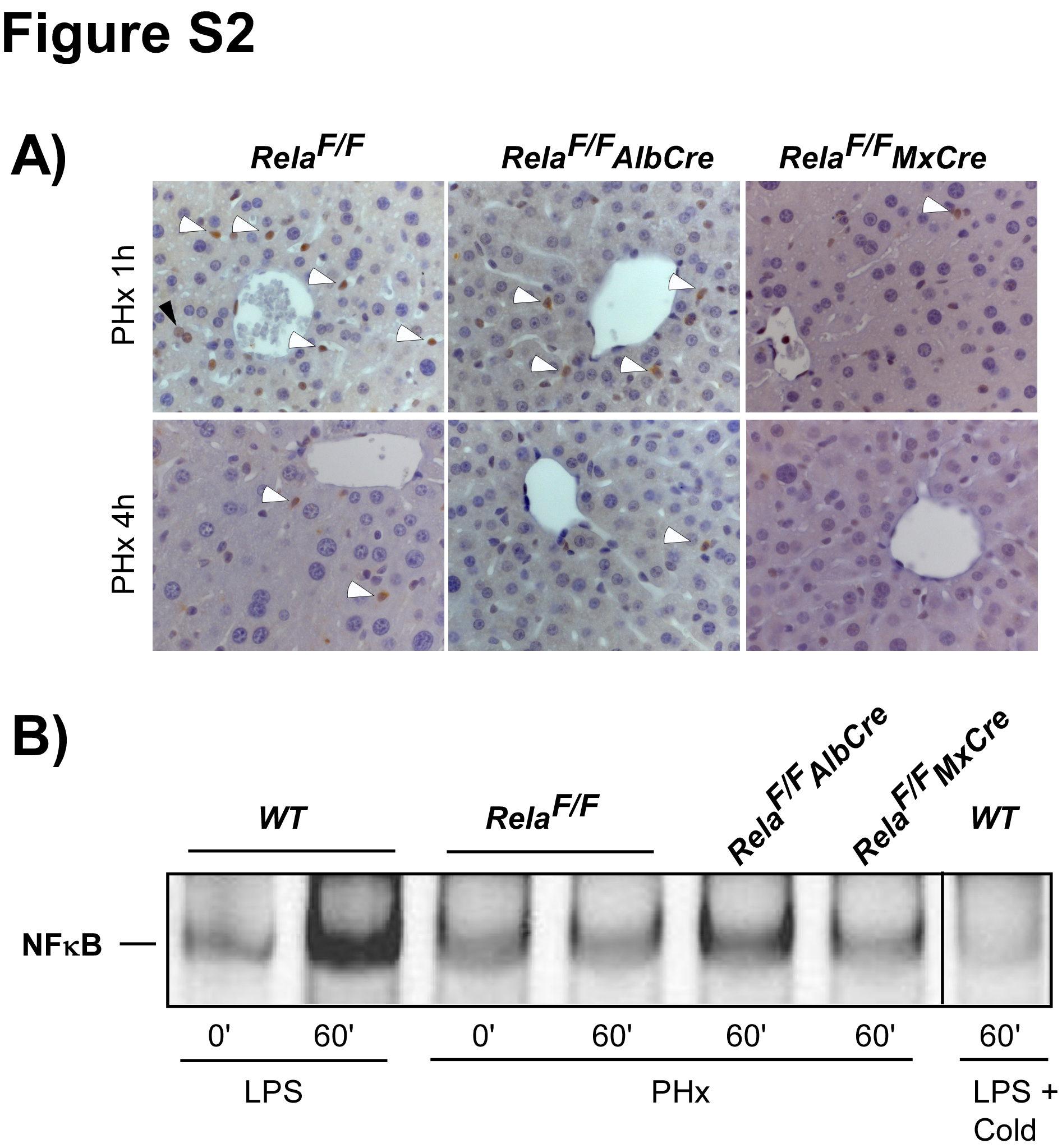

Supplement: Figure S2 — NF-κB activation after 2/3 PH. (A) NF-κB activation in livers of indicated genotypes as assessed by anti-p65 IHC at 1 h and 4 h post PH. Nuclear staining is occasionally observed in non-parenchymal cells (white arrowheads) only in control and RelaF/FAlbCre animals at 1 h and to a lesser extent at 4 h post PH. Nuclear staining for p65 is almost never detected in hepatocytes of either genotype (black arrowhead marks a positive hepatocyte in a control animal). Nuclear staining was nearly completely absent in RelaF/FMxCre animals both in hepatocytes and non-parenchymal cells (magnification×200). (B) No robust NF-κB binding activity could be detected by EMSA analysis 1 h post PH in livers of indicated genotypes. Liver lysates from LPS-treated animals served as positive control (LPS 2 mg/kg i.p.). (TIF) [file pone.0046469.s002.tif]
